# Close, but not close enough

**DOI:** 10.1007/s12471-014-0611-3

**Published:** 2014-10-10

**Authors:** J. Daemen

**Affiliations:** Erasmus Medical Center, Thoraxcenter; Room Bd-412, ’s Gravendijkwal 230, 3015 CE Rotterdam, the Netherlands

The most recent European Society of Cardiology (ESC) guidelines recommend primary percutaneous coronary intervention (PCI) as the preferred treatment for ST-segment elevation myocardial infarction (STEMI) whenever it is available within 90–120 min of the first medical contact [1]. However, timing of symptom onset appears to be hard to adjudicate precisely, and ‘ischaemic time’ thereby usually exceeds 3 h, the exact threshold after which infarct size starts to increase and long-term survival significantly starts to decline [2]. Fragmenting the different intervals of ischaemic time in the present study is therefore interesting when assessing how and where there is still room for improvement. Let us analyse the intervals in a time-dependent fashion.

First, symptom onset to diagnosis. As mentioned, precise determination of symptom onset is challenging and Postma et al. demonstrate that the interquartile range suggests times varying between 30 min and 4 to 5 h, reflecting daily clinical practice in which patients do not always directly recognise alarm symptoms and call for medical contact [3]. What is more interesting, however, is the significant difference between time of symptom onset and diagnosis, which is roughly 30 min shorter in patients picked up by ambulances with field triage capabilities versus those referred first to hospitals without primary PCI facilities (spoke hospitals). Again it is precisely this 30-minute delay that proved to result in an 8 % relative increase in 1-year mortality in STEMI patients [4].

Second, diagnosis-to-door PCI, the item in which distance to, reachability and density of PCI facilities comes into play. In Europe, first medical contact-to-balloon time ranges between 60 and 177 min, irrespective of whether the patient underwent interhospital transfer or not (approximately 50 % in Europe). In the Netherlands there is at present, with 30 facilities, a relatively close density of primary PCI centres (roughly 1/560,000 inhabitants). In European countries offering primary PCI to the majority of their STEMI patients, this population varies between 0.3 and 1.1 million per centre [5]. The density of centres offering specific and in this case complex and high-risk procedures has been the topic of debate in both the medical literature and lay press. In general, a high-volume load for operators and institutions is associated with better outcomes [6]. More specifically for primary PCI, while patients treated in high-volume centres still tend to have shorter door-to-balloon times, there is conflicting evidence on the existence of better survival rates in patients treated in high- versus lower-volume centres [7]. To assess if there is an adequate balance between volume load (and possibly outcome) and geographical spread and density of Dutch PCI sites, dedicated registries are needed to compare national outcomes with other Western countries.

Third, door-to-balloon times. Door-to-balloon times <90 min were shown to significantly decrease the incidence of major adverse cardiac events [8]. While local and nationwide programs proved to further optimise door-to-balloon times, recent literature has questioned whether further reductions will result in an additional decrease in mortality [9].

In the present study, Postma et al. assess the impact of residential distance on time to treatment in STEMI patients. The authors used a cohort of 4149 STEMI patients referred to their hospital between 2004 and 2010 and assessed whether there was a significant difference in ischaemic time in patients referred through a spoke centre or following direct triage in the ambulance, taking into account residential difference. The authors conclude that a longer distance from the patient’s residence to the PCI centre was associated with a significant increase in time to treatment, a somewhat expected finding that illustrates the importance of adequate field triage by ambulance personnel avoiding potential unnecessary referral to a spoke centre [10]. What was remarkable, however, was that in the ambulance patients, a longer residential distance to the PCI site did not lead to a longer ischaemic time although the diagnosis-to-door PCI times appeared to be 41 min in patients living within 30 km of the PCI to over 1 h in patients living 60–90 km away. Since cathlab staff are usually called in at the moment the diagnosis has been confirmed and are supposed to be up and running within 30 min, this discrepancy is hard to explain, especially since the door-to-balloon time should be added to this period increasing the average diagnosis-to-balloon time to over 90 min.

Second, the time interval between symptom onset and diagnosis in the spoke group appeared to increase with longer residential distance ‘to the PCI site’. With the density of hospitals in the Netherlands, there should almost always be one within 30 km of the patient’s residence making the delays in symptom onset to diagnosis depending on patients’ residence hard to explain.

Third, the authors hypothesised that the effect of distance on outcome might be different for spoke patients versus ambulance patients. Unfortunately, the results of the study preclude any conclusions on this question since no outcomes were reported.

A final and perhaps most questionable finding of the present study was that even after 2004, when field triage by ambulance services should have been readily available, over one-third of the STEMI population was referred to a spoke centre without primary PCI facilities. Unfortunately the data presented preclude any statements on whether this was due to lack of field triage equipment in the ambulance, which is unlikely after 2004, insufficient training of the ambulance staff or potential other confounders such as the unavailability of ECG interpreting software precluding an immediate diagnosis [10]. That adequate field triage significantly decreases ischaemic time and improves outcome does not need any further clarification. The fact that 34 % of the patients, however, were not, or not adequately triaged by ambulance services is worrying and further scrutinising of these figures might open the door to further improvement in STEMI care. Previously, Mahmoud et al. demonstrated that interhospital transfer led to a twofold increase in 1-year mortality in STEMI patients [10]. Along the same lines they found clear differences in patient characteristics between the two groups complicating the diagnosis in the spoke group (e.g. higher age, female sex and diabetes), a finding that was not observed by Postma et al.

Nevertheless, commenting on the specific results of the present study is in the end merely a detail and only relative to the scope of the problem. The authors should be applauded for providing a detailed analysis on the specific subsections of ischaemic time in their primary PCI population. Analyses like this should be an example on how logistics and quality of care in a highly developed country as the Netherlands could be monitored and reported. Especially, since it concerns a country in which the primary PCI facility density is high, rules and regulations are becoming stricter and the need for nationwide quality control registries mandated by national health care institutions is increasing. It is therefore striking to see that in a recent overview document of the ESC, in which the current STEMI care in Europe is assessed among 30 countries, national data for incidences of STEMI, in-hospital mortality, median time delays to reperfusion therapy and referral strategies are largely lacking for the Netherlands [5, 11].

At present, we are *close, but not close enough* to fulfilling the duty to report high-quality nationwide data on STEMI care. However, with several working groups and agencies such as ‘Meetbaar beter’, ‘NVVC Connect’, and the National Cardiovascular Data Registry currently joining forces to tackle this hurdle, there is light at the horizon.
